# Alterations in Respiratory Function Test Three Months after Hospitalisation for COVID-19 Pneumonia: Value of Determining Nitric Oxide Diffusion

**DOI:** 10.3390/jcm10102119

**Published:** 2021-05-14

**Authors:** Marta Núñez-Fernández, Cristina Ramos-Hernández, Francisco García-Río, María Torres-Durán, Andrés Nodar-Germiñas, Amara Tilve-Gómez, Paula Rodríguez-Fernández, Diana Valverde-Pérez, Alberto Ruano-Raviña, Alberto Fernández-Villar

**Affiliations:** 1 Service of Pneumology, University Hospital Complex of Vigo, NeumoVigo I+i. Institute of Health Research South Galicia (IISGS), 36213 Vigo, Spain; marta.nunez.fernandez@sergas.es (M.N.-F.); Cristina.Ramos.Hernandez@sergas.es (C.R.-H.); Maria.Luisa.Torres.Duran@sergas.es (M.T.-D.); 2 Service of Pneumology La Paz-IdiPAZ University Hospital, 28046 Madrid, Spain; fgrio@salud.madrid.org; 3 CIBER Respiratory Diseases (CIBERES), 28046 Madrid, Spain; 4 Department of Medicine, University Autónoma de Madrid, 28046 Madrid, Spain; 5 Infectious Diseases Unit, Service of Internal Medicine, University Hospital Complex of Vigo, 36213 Vigo, Spain; Andres.Nodar.Germinas@sergas.es; 6 Service of Radiodiagnosis, University Hospital Complex of Vigo, 36213 Vigo, Spain; Amara.Tilve.Gomez@sergas.es (A.T.-G.); Paula.Rodriguez.Fernandez@sergas.es (P.R.-F.); 7 Department of Biochemistry, Genetics and Immunology, University of Vigo, 36310 Vigo, Spain; dianaval@uvigo.es; 8 Department of Preventive Medicine and Public Health, University of Santiago de Compostela, 15704 Santiago de Compostela, Spain; alberto.ruano@usc.es; 9 Consortium for Biomedical Research in Epidemiology and Public Health (CIBERESP), 15704 Santiago de Compostela, Spain

**Keywords:** COVID-19, pneumonia, sequelae, respiratory function tests, diffusion capacity, DL_CO_, DL_NO_

## Abstract

Three to four months after hospitalisation for COVID-19 pneumonia, the most frequently described alteration in respiratory function tests (RFTs) is decreased carbon monoxide transfer capacity (DL_CO_). Methods: This is a prospective cohort study that included patients hospitalised because of SARS-CoV-2 pneumonia, three months after their discharge. A clinical evaluation, analytical parameters, chest X-ray, six-minute walk test, spirometry and DL_CO_–DL_NO_ analysis were performed. Demographic variables, comorbidities, and variables related to the severity of the admission were recorded. Results: Two hundred patients completed the study; 59.5% men, age 62 years, 15.5% admitted to the intensive care unit. The most frequent functional alteration, in 27% of patients, was in the DL_CO_–DL_NO_ combination. This alteration was associated with age, male sex, degree of dyspnoea, poorer perception of health, and limited ability for physical effort. These patients also presented higher levels of D-Dimer and more residual radiological alterations. In 42% of the patients with diffusion alterations, only reduced DL_NO_ was presented, along with lower D-Dimer levels and less capillary volume involvement. The severity of the process was associated with the reduction in DL_CO_–DL_NO_. Conclusions: The most sensitive RFT for the detection of the sequelae of COVID-19 pneumonia was the combined measurement of DL_CO_–DL_NO_ and this factor was related to patient health status and their capacity for physical exertion. In 40% of these cases, there was only a reduction in DL_NO_, a finding that may indicate less pulmonary vascular involvement.

## 1. Introduction

To date, only a limited number of studies have been published that analysed the clinical and functional alterations of patients hospitalised for SARS-COV-2 pneumonia, 3–4 months after their hospital discharge [1,2,3,4,5,6,7,8,9,10]. In these cases of severe COVID-19 pneumonia, the most frequently described alterations are a decrease in forced vital capacity (FVC), carbon monoxide transfer capacity (DL_CO_), total lung capacity, and reduction in the 6-min walk test (6MWT) [1,2,3,4,5,6,7,8,9,10]. However, the results described in these studies are not homogeneous. Although the most frequently described alteration was a decrease in DL_CO_, the reported number of patients affected by this pathology varies between 15% and 80%. Moreover, some studies define DL_CO_ according to the percentage of a reference value, while others define it in reference to the lower limit of normality (LLN) [1,2,3,4,5,6,7,8,9,10].

Publication of the first studies analysing functional alterations in patients in the first four weeks after discharge from hospitalisation with COVID-19 pneumonia [11] generated an interesting scientific debate about which value of DL_CO_ that might be used to detect functional alterations, as well as the need to adjust these values according to the alveolar volume (VA). However, more studies will be required to clarify the value of these determinations in the detection of the pulmonary sequelae of this virus [12,13]. Because patients with severe pulmonary involvement or interstitial involvement secondary to COVID-19 may present small vessel endothelial damage and altered pulmonary hemodynamic [14,15], some authors suggested that the combined determination of DL_CO_ along with the diffusion capacity of nitric oxide (DL_NO_) could help detect these vascular and interstitial changes in patients recovering after hospitalisation for these processes [16,17], as already described for other pulmonary conditions [18,19,20]. 

The combined determination of DL_CO_ and DL_NO_ is advantageous over finding the DL_CO_ alone because, in addition to providing more information, DL_NO_ testing is more stable over time and requires fewer adjustments [17,18,21]. Nonetheless, the usefulness of measuring DL_NO_ in determining the sequelae of severe COVID-19 pneumonia has only been analyzed in one study [17]. The available evidence regarding the relationship between DL_CO_ alterations and the clinical situation of the patients (e.g., dyspnoea, limitations upon physical effort or in terms of quality of life), or with other analytical parameters, imaging, or in other respiratory function tests (RFTs), is also very heterogenous. 

Therefore, we carried out this present study with the aim of identifying alterations in respiratory function in patients three months after their discharge from hospital for COVID-19 pneumonia. We determined, among other tests and functional alterations, the combined DL_CO_ and DL_NO_ levels in order to determine whether these potential alterations were related to the clinical situation of these patients and if they could be used to predict patient evolution based on their demographics or on the characteristics of the pneumonic process itself.

## 2. Materials and Methods

We included consecutive patients aged 18 and 90 years who had been discharged from a tertiary hospital between March and May 2020, after admission for COVID-19 pneumonia (confirmed by a positive PCR result for SARS-CoV-2 in the nasopharyngeal exudate or bronchoalveolar lavage). We excluded any patients who received institutional care (e.g., residences for the elderly or severely disabled), as well as any individuals who refused or were unable to sign the informed consent document. The study was approved by the Galicia Clinical Research Ethics Committee (registration number 245/2020) in April 2020. 

All patients attended a face-to-face consultation 12 ± 1 weeks after their hospital discharge, during which we performed all the planned clinical evaluations, a chest X-ray in two projections, blood tests, and a lung function study. In addition to the sociodemographic variables (age, sex, height, and weight), we also recorded the patients’ history of smoking, significant comorbidities (Table 1), their general health status before admission according to the ECOG (Eastern Cooperative Oncology Group) scale, and variables at the time of admission including arterial oxygen saturation and analytical parameters (minimum peak of total lymphocytes and maximum lactate dehydrogenase (LDH), C-reactive protein (CRP), and D-Dimer), Pneumonia Severity Index (PSI) prognosis score, unilateral or bilateral lung involvement, as well as their subsequent evolution such as intensive care unit (ICU) admission, non-invasive ventilatory support, and the total number of days of admission.

### 2.1. Clinical, Health, Radiographic, and Analytical Evaluation at 12 Weeks

The level of dyspnoea was determined according to the modified Medical Research Council (mMRC) scale. The patient health status was evaluated by applying the Spanish version of the Nottingham Health Profile (NHP) [22]. All the patients underwent a chest X-ray in two different projections; these results were reported by consensus by two expert radiologists blinded to the clinical history, who compared them with the studies from the time of discharge and from six months prior to hospital admission (if available); these results were reported either as the complete resolution of the lesions (normal study or similar to the one existing prior to admission) or as incomplete resolution. The values for total lymphocytes, LDH, CRP, D-Dimer, and the N-terminal portion of pro-natriuretic peptide type B (NT-proBNP) were determined from the blood samples. 

### 2.2. Respiratory Function Tests at 12 Weeks

The RFTs were conducted with MasterScreen PFT equipment (Viasys, CareFusion, Würzburg, Germany) with SentrySuite^TM^ software and included a forced spirometry and bronchodilator test, following the recommendations of the American Thoracic Society and European Respiratory Society (ATS/ERS) [23] and using the values provided by the Global Lung Function Initiative (GLI) [24] equations as reference values. DL_NO_ and DL_CO_ were measured simultaneously during a single breath maneuver using the above-mentioned equipment, according to ERS recommendations [21]. After maximal expiration, the patients were requested to inhale quickly and deeply in less than four seconds a gas mixture of 0.3% CO, 9.0% He, 21% O2 and 400 ppm NO in N2 and inhaled from a plastic bag containing a final concentration of NO of 50 ppm obtained ≤2 min before its use. The pressure curve displayed during the occlusion showed whether the patient has held his/her breath and the maneuver was accepted when pressure was <3 kPa. A breath-hold of 6 s was then requested, followed by a rapid expiration. The first 0.75 L of expired gas was rejected and the following 0.75 L was sampled in a bag, which was automatically analysed for NO, CO and He. This washout volume was 0.5 L for subjects with a vital capacity <2 L. The actual breath-hold time was calculated using the Jones and Meade method [25]. The linearity of the electrochemical cell was checked by factory and the apparatus was calibrated for gas fractions using automated procedures. The procedure was repeated after a 4-min wait, and it was accepted if two successive DLNO and DLCO measurements were within 17 and 3 mL/min/mmHg, respectively. If this was not the case, additional measurements (up to five in total) were performed. The mean of two chosen manoeuvres was used for the subsequent analyses. 

The inert gas, He, was used in the calculation of alveolar volume (VA) by means of the He-dilution technique. The values of the membrane component of diffusing capacity for CO (DMCO) and of the pulmonary capillary blood volume (Vc) were calculated ac-cording to the model by Guénard et al. [26]. All diffusing capacity values were corrected for the haemoglobin levels obtained on the same day of the study and interpreted ac-cording to the reference equations proposed by Zavorsky et al. [21].

The 6MWT was performed in duplicate, along a 30 m corridor, following the ATS recommendations [27]. The oxygen saturation and scores on the Borg dyspnoea scale were recorded before starting and at the end of the 6MWT, and the differences and total distance walked were calculated. 

### 2.3. Statistical Analysis

The quantitative variables were expressed as the median and 25% and 75% percentiles. Qualitative variables were expressed by their number and percentage, except for the percentage of RFT alterations, for which we calculated the 95% confidence interval (95% CI). Numerical variables were compared using the Mann–Whitney U test and qualitative variables were compared using Chi-squared or Fisher exact tests. Comparison of the frequencies of the different functional alterations was carried out using the McNemar test and the degree of agreement was compared using Cohen’s kappa coefficient. 

A comparative analysis of the demographic and clinical variables prior to admission and was carried out. We also compared the severity of the pneumonic process among patients with RFT alterations to the variables most frequently affected and to the clinical situation variables (dyspnoea, physical exertion capacity, impact of exertion, and global health status) at 12 weeks. To evaluate possible relationships independently of the variables related to DL_NO_ alteration in the univariate analysis, a conditional forward logistic regression model was constructed, which included all the variables that had obtained a *p* < 0.05 in the univariate analysis, calculating the odds ratios (ORs) and their 95% CIs. SPSS software for Windows (version 25; IBM Corp, Armonk, NY, USA) was used in all analyses. 

## 3. Results

We consecutively evaluated 225 patients; 129 (57.3%) were male and the mean age was 62 (50–71) years. Of the total cohort, 207 performed a valid and reproducible forced spirometry, while 198 completed the bronchodilator test, and 200 completed the DL_NO_–DL_CO_ manoeuvres and 6MWT. The main characteristics of the patients who met the inclusion criteria are summarised in Table 1.

Table 2 describes the RFTs results. The most frequent alteration in RFTs was a reduction in DL_CO_ (<LLN), which was observed in 58 (29%) of the patients and was significantly higher than all the other tests performed (*p* < 0.001); 24 patients (12%) had a DL_NO_ < LLN but with a DL_CO_ > LLN, and both these figures were altered in 34 (17%) of them. All patients with DL_CO_ < LLN also presented DL_NO_ < LLN. The concordance between the alterations in the different spirometry and diffusion variables is shown in Table 3; the only high values were between DL_NO_ and DL_CO_, DL_NO_ and DM_CO,_ and K_NO_ and K_CO_.

Table 4 shows the results for the patient clinical situation, health status, and capacity for physical exertion (as determined using the 6MWT) 12 weeks after discharge, both for the entire sample and dichotomised according to whether the patient DL_NO_ was lower or higher than the LLN. Patients with a reduced DL_NO_ presented a higher level of dyspnoea, and a greater number of altered health status dimensions (especially at the expense of physical mobility and social isolation), and a lower capacity for physical exertion, which more strongly impacted their oxygen saturation. They also had higher D-Dimer values and the lesions on their chest radiographs persisted for longer. Furthermore, 34 of the 58 patients who had a reduced DL_NO_ also showed a decreased DL_CO_. 

Table 5 compares these same variables depending on whether the functional alteration was detected only DL_NO_, or in both DL_CO_ and DL_NO_. Proportionally more patients with an altered DL_NO_ but not an altered DL_CO_ were younger females. The presence or absence of a reduction in DL_CO_ in patients with decreased DL_NO_ did not discriminate between individuals with different levels of dyspnoea, health status, tolerance to physical exertion, radiological resolution, or laboratory disorders, except for D-dimer levels, which were higher in patients with decreased DL_CO_ and reduced DL_NO_. The Vc < LLN in 1/24 (4.2%) patients with a reduced DL_NO_ but normal DL_CO_ versus 16/34 (47.1%) with a DL_NO_ and reduced DL_CO_ (*p* = 0.0004).

Among the patients with a DL_NO_ < LLN, those with an altered DL_NO_ but DL_CO_ > LLN presented significant differences (*p* < 0.05) in terms of mobility, the social dimensions of the NHP, and both in the drop in oxygen saturation during the 6MWT and the final oxygen saturation afterwards (Table 5).

Table 6 shows the patient demographic and clinical factors prior to admission and the variables recorded that were related to the severity of the SARS-CoV-2 pneumonic process. Only three cases of pulmonary embolism were described. In the univariate analysis, both age and male sex, as well as the presence of various comorbidities and the general condition of the patient were related to a higher frequency of DL_NO_ alterations. Similarly, variables related to the severity of the pneumonic process, such as a higher PSI scale score, need for non-invasive ventilatory support, admission to the ICU, lower oxygen saturation upon admission, or the peak levels reached for parameters such as total lymphocytes, CRP, LDH, or D-Dimer, also showed significant differences. 

In the multivariate analysis, male sex (OR = 6; 95CI% = 1.7–20; *p* = 004), age (OR = 0.93; 95CI% = 0.89–0.98; *p* = 0.009), days of admission (OR = 1.03; 95CI% = 1.01–1.06; *p* = 0.009), and the PSI score (OR = 1.07; 95CI% = 1.03–1.1; *p* = 0.0001) were independently related to the presence of a reduced DL_NO_ 12 weeks after hospital discharge.

## 4. Discussion

This study provides novel information because it is one of the first to include an analysis of DL_NO_ among the RFTs performed three months after discharge of patients hospitalised for SARS-CoV-2 pneumonia. One of the main conclusions of this work is that the DL_NO_ lung function test results are most frequently altered in these patients, with this alteration present in almost double the number of patients with decreased DL_CO_ (currently the most studied diffusion test in this context). Altered DL_NO_ was related to the degree of dyspnoea three months after discharge; it was also associated with the persistence of lesions on patient radiographs and affected these patients’ health status and capacity for physical exertion. Age and sex, as well as the severity of the process, helped to predict this functional alteration. 

Although this technique is not routinely used in clinical practice, the evidence available for various diffuse cardiopulmonary pathologies indicates that evaluating pulmonary diffusion using the combination of DL_NO_ and DL_CO_ is more sensitive than evaluating DL_CO_ alone [17,18,19,20]. Some 40% of the patients with a decreased DL_NO_ presented a DL_CO_ within normal limits, with a higher proportion of women and younger patients in this group. The impact of COVID-19 pneumonia on the health and limitations in terms of physical exertion in these patients was higher than those without diffusion alterations. 

While both the DL_NO_ and DL_CO_ techniques depend on the same components (membrane conductance and the vascular component), DL_CO_ is more sensitive to microvascular alterations while DL_NO_ is more influenced by the membrane component of diffusion [17,18,21]. The fact that one of the possible pulmonary effects of COVID-19 is small vessel microthrombosis [28] could perhaps explain the elevated D-Dimer levels we found in patients with combined alterations in both DL_CO_ and DL_NO_, as well as the different intensity of Vc involvement compared to patients in which only DL_NO_ was altered_._ This finding could perhaps also explain the persistent symptoms present in many of these patients in whom no alterations were observed in conventional pulmonary function tests, including DL_CO_. However, it is important to consider that the association between the persistence of symptoms, their severity, and alterations in pulmonary function tests is variable in work published to date [29].

Notwithstanding, this current work reveals the limited concordance between many other RFTs, which may reflect the complexity of the structural involvement in patients who have suffered severe COVID-19 pneumonia. These patients may suffer persistent lesions at the level of the parenchyma, distal airways, and small pulmonary vessels (as endothelitis or microthrombosis), as well as haemodynamic alterations that can compromise the ventilation/perfusion balance, all for varying amounts of time [12,13,14,15,16,17]. In addition, patients’ own underlying diseases, the frequent need for high oxygen concentrations for several days (with the consequent risk of hyperoxia), interrelation between different functional parameters (such as the influence of the VA), and variability in performance of each evaluation technique, may also influence the heterogeneity seen in the work published so far [12,13,14,15,16,17,30]. 

In our opinion, the first priority for the RFTs used to assess the impact of COVID-19 infection must correlate well with the symptoms and limitations presented by the patient and thus, be able to explain them and thereby avoid the unnecessary search for other possible aetiologies (such as cardiac, muscular, or psychological alterations). Most importantly, these tests must be able to reliably predict functional alterations based on variables that are easy to register (either from the patient or according to the severity of the process) because this will allow physicians to prioritise interventions and initiate preventive and rehabilitative strategies early on [31]. 

Based on the results of our study, the DL_NO_ test best meets these previously mentioned criteria. Measurement of the different components of diffusion can allow earlier and more precise identification of microvascular alterations. Barisione and Brusasco have recently published that these alterations evolve differently in the longer term [17]. Moreover, there are some technical advantages to DL_NO_ over conventional DL_CO_, such as the fact that it is independent from haemoglobin and lung volumes, which can be important in patients with comorbidities and the sequelae of severe respiratory disease.

The scarcity of previous studies evaluating DL_NO_ makes it difficult to contextualise this work within the existing academic literature. Furthermore, the few series that have been published regarding functional alterations detected three-to-four months after a COVID-19 pneumonic episode mainly examined the DL_CO_ [1,2,3,4,5,6,7,8,9,10]. As shown in the table summarising these studies (Table 7), and despite the fact that there is little variation in the percentage over the reference value of DL_CO_, when the alteration was defined by a decrease under 80% in the theoretical percentage (51–57%), except for series that include only post-ICU patients [10]; however, when this was measured as a decrease below the LLN, DL_CO_ alteration ranged from 17% in our work to 24% as reported by Lerum et al. [1], and 34% in another study nine weeks after discharge [32]. 

In other studies, the relationship between predictor variables and lung involvement was enhanced by different linear correlation analyses without defining specific cut-off points for the definition of DL_CO_ alteration [6,8].

These different definitions can influence the capacity of DL_CO_ to predict the functional sequelae of the different variables analysed. However, as we observed in this present work, and consistent with the results from the only published study that evaluated patients six months after infection with SARS-CoV-2 [33], these variables are usually associated with parameters related to the severity of the pneumonic episode (e.g., prognostic scales, value of analytical determinations indicative of greater inflammation) and intensity of the supportive therapy required during patient admission [1,2,3,4,5,6,7,8]. One study found that cardiorespiratory comorbidities and female sex predicted DL_CO_ < 80%, but not DL_CO_ < 60% [7]. However, other studies found that men tended to be affected more often [2], which agrees with our series in which the male sex was a powerful predictive factor of decreased DL_CO_. Although this factor requires more research, male sex was a predictor for the long-term development of fibrosis in other coronavirus infections such as SARS [34].

The main limitation of this study was that it was carried out in a single centre and the number of patients who required admission to the intensive care unit was lower than in other studies It should be taken into account when interpreting the results. However, the general characteristics of the patients we included, as well as the values of the variables analysed in the series published so far, are relatively superimposable, meaning that it is possible to extrapolate our results to other centres and countries.

In addition, we did not systematically include data from more sophisticated imaging techniques such as chest computed tomography or echocardiography that could have helped better define the degree of pulmonary involvement or the coexistence of other cardiovascular diseases. Also, as in the other studies published so far, the value of lung function tests prior to COVID-19 was not available and we have not excluded patients with a history of smoking or with cardiovascular comorbidity, which could influence the results.

We consider the strengths of our work to be the fact that we consecutively recruit patients in a tertiary hospital which provides health coverage to 95% of the reference population, an acceptable casuistry compared to other published work. We have collected a remarkable number of variables that we analysed at three months and it is one of the few published studies that includes the determination of DLNO among lung function tests. We used definitions based on recommendations by scientific societies, and all tests were performed by the same professionals with more than 10 years of experience (thereby helping to reduce variability in the results).

## 5. Conclusions

Combined determination of DL_CO_ and DL_NO_ was the most sensitive test to evaluate the medium-term sequelae of severe COVID-19 pneumonias in our patient cohort. Altered DL_CO_–DL_NO_ was related to the severity of patient symptoms and their health status and could be predicted by sociodemographic factors and the severity of the pneumonic process. These findings may be useful for evaluating patients with persistent symptoms who do not present alterations in commonly used RFTs. Similar studies will be required to increase the evidence in this field and to improve our knowledge of the possible sequelae of lung involvement in COVID-19 and its evolution over time [17].

## Figures and Tables

**Table 1:** Sociodemographic, clinical, and general characteristics of the pneumonic process caused by COVID-19 in the patients included in the study.

Variables	Total *N* = 200
**Demographics and Clinics before Admission**	
Male sex, *N* (%),	119 (59.5)
Age, years	62 (50–71)
Body mass index (Kg/m^2^)	28.7 (25.9–31.9)
Previous and current smoker, *N* (%)	84 (42)
History of chronic cardiopathy, *N* (%) *	37 (18.5)
History of diabetes, *N* (%)	23 (11.5)
History of hypertension, *N* (%)	74 (37)
History of COPD, *N* (%)	6 (3)
History of chronic renal failure, *N* (%)	7 (3.5)
ECOG score	1 (1–2)
ECOG score ≥ 2, *N* (%)	51 (25.5)
**In Relation to the Pneumonic Process**	
Bilateral radiographic involvement, *N* (%)	139 (69.5)
Oxygen saturation at hospital admission	96 (94–98)
Pneumonia Severity Index	61 (49–75)
Pneumonia Severity Index ≥ 3, *N* (%)	62 (31)
Need non-invasive ventilatory support, *N* (%)	12 (6)
ICU admission, *N* (%)	31 (15.5)
Lowest level of lymphocites (10^9^/L)	0.75 (0.55–1.04)
Maximum level of C-reactive protein (mg/L)	83.3 (31–160.1)
Maximum level of LDH (U/L)	295 (227.7–386.7)
Maximum level of D-dimer (ng/mL) **	1150.1 (476.2–2856)
Length of stay (days)	7 (4–13)

* History of ischaemic heart disease or chronic heart failure; ** Available in 94 patients only; COPD: Chronic obstructive pulmonary disease; ECOG: Eastern Cooperative Oncology Group; ICU: Intensive care unit.

**Table 2:** Results of respiratory function tests at 12 weeks.

**Spirometry**	**Values and Frequencies**
FVC, L	3.62 (3.02–4.32)
FVC, % of predicted	103 (92.5–114.7)
FVC < LLN, *N* (% (IC 95%))	9 (4.3 (1.5–7.1))
FEV_1_, L	2.9 (2.43–3.45)
FEV_1_, of predicted	104 (95.2–114)
FEV_1_ < LLN, *N* (% (IC 95%))	9 (4.3 (1.5–7.1))
FEV_1_/FVC (%)	79 (75–84)
FEV_1_/FVC < LLN, *N* (% (IC 95%))	9 (4.3 (1.5–7.1))
**Post-broncodilation Changes**	**Values and Frequencies**
FVC, L	0.680 (0.52–0.720)
FVC, %	1 (−2–5)
FEV_1_, L	0.120 (0.55–0.210)
FEV_1_, %	4 (2–8)
Positive bronchodilator test, *N* (% (IC 95%))	13 (6.5 (3–10.1))
**Gas Diffusion**	**Values and Frequencies**
DL_CO_, mL/min/mmHg	21.2 (16.4–25.7)
DL_CO_, % of predicted	84 (74–97)
DL_CO_ < LLN, *N* (% (IC 95%))	34 (17 (11.7–22.1))
K_CO_, mL/min/mmHg/L	4.3 (3.7–4.8)
K_CO_, % of predicted	96 (84–105)
K_CO_ < LLN, *N* (% (IC 95%))	13 (6.5 (3.1–9.9))
DL_NO_, mL/min/mmHg	99.1 (73.1–115.5)
DL_NO_, % of predicted	76.5 (68.2–87.0)
DL_NO_ < LLN, *N* (% (IC 95%))	58 (29 (22.7–35.3))
K_NO_, mL/min/mmHg/L	19.3 (16.6–21.6)
K_NO_, % of predicted	89.7 (83–99)
K_NO_ < LLN, *N* (% (IC 95%))	29 (10.5 (6.2–14.8))
DM_CO_, mL/min/mmHg	81.5 (59–99.7)
DM_CO_, % of predicted	69 (57–80)
DM_CO_ < LLN, *N* (% (IC 95%))	58 (29 (22.7–35.3))
VC, mL	54.3 (44–65)
VC, % of predicted	85 (74–97.7)
VC < LLN (*N*, % (IC 95%))	20 (10 (5.8–14.2))
VA, L	4.9 (4.2–5.7)
VA, % of predicted	87 (77.2–94)
VA < LLN, *N* (% (IC 95%))	41 (20.5 (14.9–26.1))

DL_CO_: diffusion capacity of carbon monoxide; DL_NO_: diffusion capacity of nitric oxide; DM_NO_: membrane conductance of nitric oxide; K_CO_: Rate of uptake of carbon monoxide from alveolar gas; K_NO_: Rate of uptake of nitric oxide from alveolar gas; FEV_1_: forced expiratory volume in first one second; FVC: forced vital capacity; LLN: lower limits of normal; VA: alveolar lung volume; VC: pulmonary capillary blood volume.

**Table 3:** Concordance between the alteration between the different functional tests performed, including the coincident number and the kappa coefficient (in parentheses).

	DL_CO_ < LLN *N* = 34	K_CO_ < LLN *N* = 13	K_NO_ < LLN *N* = 29	VA < LLN *N* = 8	DM_CO_ < LLN *N* = 58	VC < LLN *N* = 20	FVC < LLN *N* = 9	FEV_1_ < LLN *N* = 9
DL_NO_ < LLN *N* = 58	34 (0.67)	12 (0.26)	17 (0.32)	31 (0.51)	47 (0.73)	17 (0.34)	6 (0.53)	8 (0.56)
DL_CO_ < LLN *N* = 34		11 (0.41)	14 (0.43)	22 (0.49)	30 (0.55)	16 (0.53)	4 (0.15)	5 (0.18)
K_CO_ < LLN *N* = 13			13 (0.74)	9 (0.26)	10 (0.19)	9 (0.51)	2 (0.16)	3 (0.25)
K_NO_ < LLN *N* = 29				10 (0.21)	15 (0.27)	11 (0.49)	2 (0.09)	4 (0.23)
VA < LLN *N* = 8					32 (0.53)	13 (0.28)	7 (0.25)	7 (0.23)
DM_CO_ < LLN *N* = 58						12 (0.19)	5 (0.10)	6 (0.12)
VC < LLN *N* = 20							4 (0.26)	5 (0.32)
FVC < LLN *N* = 9								4 (0.51)

DL_CO_: diffusion capacity of carbon monoxide; DL_NO_: diffusion capacity of nitric oxide; DM_NO_: membrane conductance of nitric oxide; K_CO_: Rate of uptake of carbon monoxide from alveolar gas; K_NO_: Rate of uptake of nitric oxide from alveolar gas; FEV1: forced expiratory volume in first one second; FVC: forced vital capacity; LLN: lower limits of normal; VA: alveolar lung volume; VC: pulmonary capillary blood volume.

**Table 4:** Degree of dyspnoea, health status, capacity for physical exertion, analytical determinations, and radiological resolution at 12 weeks, according to whether DL_NO_ < LLN or not.

Variables	Total *N* = 200	DL_NO_ > LLN *N* = 142	DL_NO_ < LLN *N* = 58	*p*
**Dyspnoea According to mMRC**				
Dyspnoea ≥ 1, *N* (%)	89 (44)	55 (38.7)	34 (58.6)	0.01
Dyspnoea ≥ 2, *N* (%)	24 (12)	12 (8.4)	12 (20.6)	0.01
Dyspnoea	0.0 (0–1)	0 (0–1)	1 (0–1)	0.001
**Health Status According to Nottingham Health Profile**				
Energy	0 (0–33.3)	0 (0–33.3)	0 (0–33.3)	0.12
Pain	12.5 (0–37.5)	12.5 (0–37.5)	12.5 (0–62.5)	0.15
Physical mobility	12.5 (0–37.5)	12.5 (0–37.7)	25 (0–50)	0.02
Emotional reactions	11.1 (0–33.3)	11.1 (0–22.2)	11.1 (0–36.1)	0.21
Sleep	20 (0–60)	20 (0–60)	20 (0–80)	0.61
Social isolation	0 (0–0)	0 (0–0)	0 (0–20)	0.02
Number of limited areas	3 (2–4)	3 (1–4)	3.5 (2–5)	0.04
**Exercise Capacity (6MWT)**				
6-min walking distance, m	457 (379.5–520.1)	473 (416–532)	411 (369.2–474)	0.04
Initial oxygen saturation, %	98 (97–99)	98 (97–99)	98 (97–98)	0.02
Final oxygen saturation, %	97 (95–98)	97 (96–98)	96 (94–97)	0.001
Oxygen saturation drop, %	1 (1–3)	1 (0–2)	2 (1–4)	0.001
Borg Scale Dyspnoea Start (1–10)	0 (0–1)	0 (0–1)	0 (0–1)	0.17
Final Borg scale dyspnoea (1–10)	2 (0–4)	2 (0–4)	1 (0–4)	0.75
**Laboratory Parameters**				
Lymphocytes x10^9^/L	1.8 (1.4–2.2)	1.7 (1.4–2.2)	1.8 (1.4–2.3)	0.43
C-reactive protein, mg/L	1.2 (0.4–3.1)	1 (0.4–3)	1.7 (0.5–3.1)	0.07
D-dimer, ng/mL	337 (225–565)	317 (217–533)	454 (263–791)	0.02
Lactate dehydrogenase, U/L	195 (173–216)	193 (172–214)	201 (181.2–221.5)	0.10
Ferritin, ng/mL	68 (29–136)	74 (27–125.5)	60 (29–145.2)	0.98
NT-proBNP, pg/mL	73 (37.7–167.2)	74 (38.5–149.0)	80 (34.5–204)	0.39
**Chest X-ray**				
Persistence of any lung injuries, *N* (%)	52 (26)	22 (15.5)	30 (51.7)	0.001

6MWT: Six minutes walking test; LLN: lower limits of normal; mMRC: Modified Medical Research Council; X-ray: Radiography.

**Table 5:** Demographic variables, degree of dyspnoea, health status, capacity for physical exertion, analytical determinations, and radiological resolution at 12 weeks, according to the presence or absence of alterations in DL_NO_, or DL_NO_ and DL_CO._

Variables	DL_NO_ < LLN y DL_CO_ > LLN *N* = 24	DL_NO_ < LLN y DL_CO_ < LLN *N* = 34	*p*
**Demographics**			
Male sex, *N* (%),	20 (83.3)	34 (100)	0.01
Age, years	60 (51–69)	68 (61.5–74)	0.02
**Dyspnoea According to mMRC**			
Dyspnoea ≥ 1, *N* (%)	12 (50)	22 (64.7)	0.20
Dyspnoea ≥ 2, *N* (%)	3 (12.5)	9 (26.4)	0.19
Dyspnoea	0.5 (0–1)	1 (0–1)	0.06
**Health Status According to Nottingham Health Profile**			
Energy	0 (0–33.3)	0 (0–33.3)	0.88
Pain	6.2 (0–59.3)	12.5 (0–62)	0.51
Physical mobility	31.2 (0–50)	25 (0–50)	0.75
Emotional reactions	11.1 (0–44.4)	11.1 (0–22.6)	0.20
Sleep	30 (0–80)	20 (0–80)	0.99
Social isolation	0 (0–35)	0 (0–20)	0.19
Number of limited areas	3 (2–5)	4 (2–5)	0.84
**Respiratory Function Tests**			
FVC, % of predicted	95 (84–106.2)	89.9 (84–97.5)	0.16
FEV_1_, % of predicted	94.5 (86.1–105.2)	86.7 (80.7–99)	0.31
DL_CO_, % of predicted	76 (72.2–80)	55.3 (48.5–65.5)	0.0001
DL_NO_, % of predicted	69 (62–70)	51.2 (43–63)	0.0001
K_CO_, % of predicted	96 (84–106.2)	76.3 (68.7–86)	0.0001
K_NO_, % of predicted	89 (81.2–94)	74.4 (65–83.5)	0.0001
DM_CO_, % of predicted	56 (50.2–61.7))	42.5 (33.5–56.2)	0.0001
VC, % of predicted	81 (72.2–88.7)	64 (33.5–56.2)	0.0001
VA, % of predicted	79 (74–87)	72.5 (62.7–84.2)	0.13
**Exercise Capacity (6MWT)**			
6-min walking distance, m	450 (386–509)	434 (347.5–462.7)	0.30
Initial oxygen saturation, %	98 (97–98)	98 (97–98)	0.67
Final oxygen saturation, %	96 (95–97)	95 (92.7–97.2)	0.52
Oxygen saturation drop, %	2 (0.2–3)	2 (1–4.2)	0.30
Borg Scale Dyspnoea Start (1–10)	0 (0–1)	0 (0–0)	0.28
Final Borg scale dyspnoea (1–10)	1.5 (0.2–5.5)	1 (0–4)	0.33
**Laboratory Parameters**			
Lymphocytes × 10^9^/L	2 (1.4–2.5)	1.7 (1.3–2.3)	0.42
C-reactive protein, mg/L	1.7 (0.9–3)	1.8 (0.4–5)	0.94
D-dimer, ng/mL	295 (215.2–523.7)	510 (371.5–954.5)	0.004
Lactate dehydrogenase, U/L	204 (185–222.5)	199 (171.5–218.7)	0.42
Ferritin, ng/mL	55 (21.2–154.2)	83 (31.2–145.2)	0.92
NT-proBNP, pg/mL	76 (22–157.7)	116 (52–375)	0.16
**Chest X-ray**			
Persistence of any lung injuries, *N* (%)	13 (53.2)	15 (44.1)	0.45

6MWT: Six minutes walking test; DL_CO_: diffusion capacity of carbon monoxide; DL_NO_: diffusion capacity of nitric oxide; DM_NO_: membrane conductance of nitric oxide; K_CO_: Rate of uptake of carbon monoxide from alveolar gas; K_NO_: Rate of uptake of nitric oxide from alveolar gas; FEV1: forced expiratory volume in first one second; FVC: forced vital capacity; LLN: lower limits of normal; VA: alveolar lung volume; VC: pulmonary capillary blood volume.

**Table 6:** Predictive factors for a decrease in DL_NO_ in the univariate analysis.

Variables	DL_NO_ > LLN *N* = 142	DL_NO_ < LLN *N* = 58	*p*
**Demographics and Clinical before Admission**			
Male sex, *N* (%),	65 (45.8)	54 (93.1)	0.0001
Age, years	61 (46.7–70)	65,5 (56.7–73)	0.03
Body mass index (Kg/m^2^)	28.7 (25.4–32.3	28.1 (26.6–31.7)	0.59
Previous and current smoker, *N* (%)	47 (33.1)	37 (63.8)	0.001
History of chronic cardiopathy, *N* (%)	20 (14.1)	17 (29.3)	0.01
History of diabetes, *N* (%)	11 (7.7)	12 (20.7)	0.01
History of hypertension, *N* (%)	47 (33.1)	27 (46.6)	0.07
History of COPD, *N* (%)	1 (0.7)	5 (8.6)	0.003
History of chronic renal failure, *N* (%)	3 (2.1)	4 (6.9)	0.11
ECOG score	1 (1–1)	1 (1–2)	0.01
ECOG score ≥ 2, *N* (%)	29 (20.4)	22 (37.9)	0.01
**In Relation to the Pneumonic Process**			
Bilateral radiographic involvement, *N* (%)	39 (67.2)	100 (70.4)	0.65
Oxygen saturation at hospital admission	96.5 (94–98)	94.9 (91.5–96)	0.0001
Pneumonia Severity Index	57 (43–67.5)	74 (62–93.5)	0.0001
Pneumonia Severity Index ≥ 3, *N* (%)	30 (21)	32 (56)	0.0001
Need non-invasive ventilatory support, *N* (%)	5 (3.5)	7 (12.1)	0.02
ICU admission, *N* (%)	14 (9.9)	17 (29.3)	0.001
Lowest level of lymphocites (10^9^/L)	0.82 (0.59–1.18)	0.60 (0.42–0.81)	0.0001
Maximum level of C-reactive protein (mg/L)	62.4 (23.6–138.5)	148.6 (88.6–217.7)	0.0001
Maximum level of lactate dehydrogenase (U/L)	277 (219.5–343.2)	367.5 (265.2–464.2)	0.005
Maximum level of D-dimer (ng/mL) *	690.1 (423–2221.7)	1881.5 (980–4018)	0.001
Length of stay (days)	8.8 (3–11)	10.5 (6–26)	0.0001

* Available in 94 patients only; COPD: Chronic obstructive pulmonary disease; DL_CO_: diffusion capacity of carbon monoxide; DL_NO_: diffusion capacity of nitric oxide; DM_NO_: membrane conductance of nitric oxide; ECOG: Eastern Cooperative Oncology Group; K_CO_: Rate of uptake of carbon monoxide from alveolar gas; K_NO_: Rate of uptake of nitric oxide from alveolar gas; FEV1: forced expiratory volume in first one second; FVC: forced vital capacity; ICU: Intensive care unit; LLN: lower limits of normal; VA: alveolar lung volume; VC: pulmonary capillary blood volume.

**Table 7:** Comparison of the principal variables of the main published series that had evaluated pulmonary function tests three-to-four months after patient hospitalisation for COVID-19 pneumonia.

Authors and Reference	Lerum [1] 2020	Shah [3] 2020	Qin [2] 2021	Sibila [3] 2021	González [10] 2021	Anastasio [8] 2021	Belan [7] 2021	Guler [6] 2021	
Country	Norway	Canada	China	Spain	Spain	Italy	Italy	Switzerland	
Time from hospital discharge, months	3	3	3	3	3	4	4	4	
*N*	103	60	88	172	62	222	219	47 Mild	66 Severe
Male sex, %	52	68	42	57	74.2	57.2	59.7	52.9	60
Age, years	59	67	59	56.1	60	58	61	57.4	60.3
BMI	25.8	25	23.8	-	28.2	26.2	-	25.5	29.8
Current or previous smoking, %	36	38	-	27.3	56.7	37.9	41.1	37	56
ICU admission, %	15	-	-	43	100	15.3	11.8	-	
Length of stay (days)	6	10		-	-	-		-	
Diabetes, %	8	22	9	14.5	14.5	9.5	15.1	0	35
COPD or asthma %	-	13	-	11.6	9.6	9.1	5.8	22	21
Hypertension, %	35	35	28	33.1	37.1	39.6	41.2	8	55
Cardiopathy, %	-	10	9	13.9	9.4	15.3	16.3	9	10
Dyspnoea mMRC > 0	54	20		48.8	46.7	48.2	5.5	-	-
Dyspnoea mMRC > 1	-	-		-	-	20.7	-	-	-
Chest CT	Yes	Yes	-	No	Yes	No	No	Yes	
FVC, %	94	94	89.7	90	81.5	105	98.5	95.6	86.6
FEV_1_, %	92	93	92.9	94	88.9	106	101	94	89.4
DL_CO_, %	83	77	82-6	-	-	-	79	95.3	72.2
DL_CO_ < LLN, %	24	-	-	-	-	-	-	-	-
DL_CO_ < 80%, %	-	52	54	57	82	-	51.6	-	-
DL_CO_/VA, %	95	-	86.1	-	-	101	-	-	-
DL_CO_/VA < 80%			38	-	.				
6-min walking distance, m	580	504	-	-	400	500	-	576	456

BMI: body mass index; COPD: chronic obstructive pulmonary diseases; CT: computerized tomography; DL_CO_: diffusion capacity of carbon monoxide; FEV1: forced expiratory volume in first one second; FVC: forced vital capacity; LLN: lower limits of normal; VA: alveolar lung volume.
